# Supplementary figures and images for: Titin-Truncating variants predispose to dilated cardiomyopathy in populations genetically similar to african and european reference populations
Source: PLoS Genet. 2025 Jun 13;21(6):e1011727. doi: 10.1371/journal.pgen.1011727 (PMC12237270; doi:10.1371/journal.pgen.1011727)

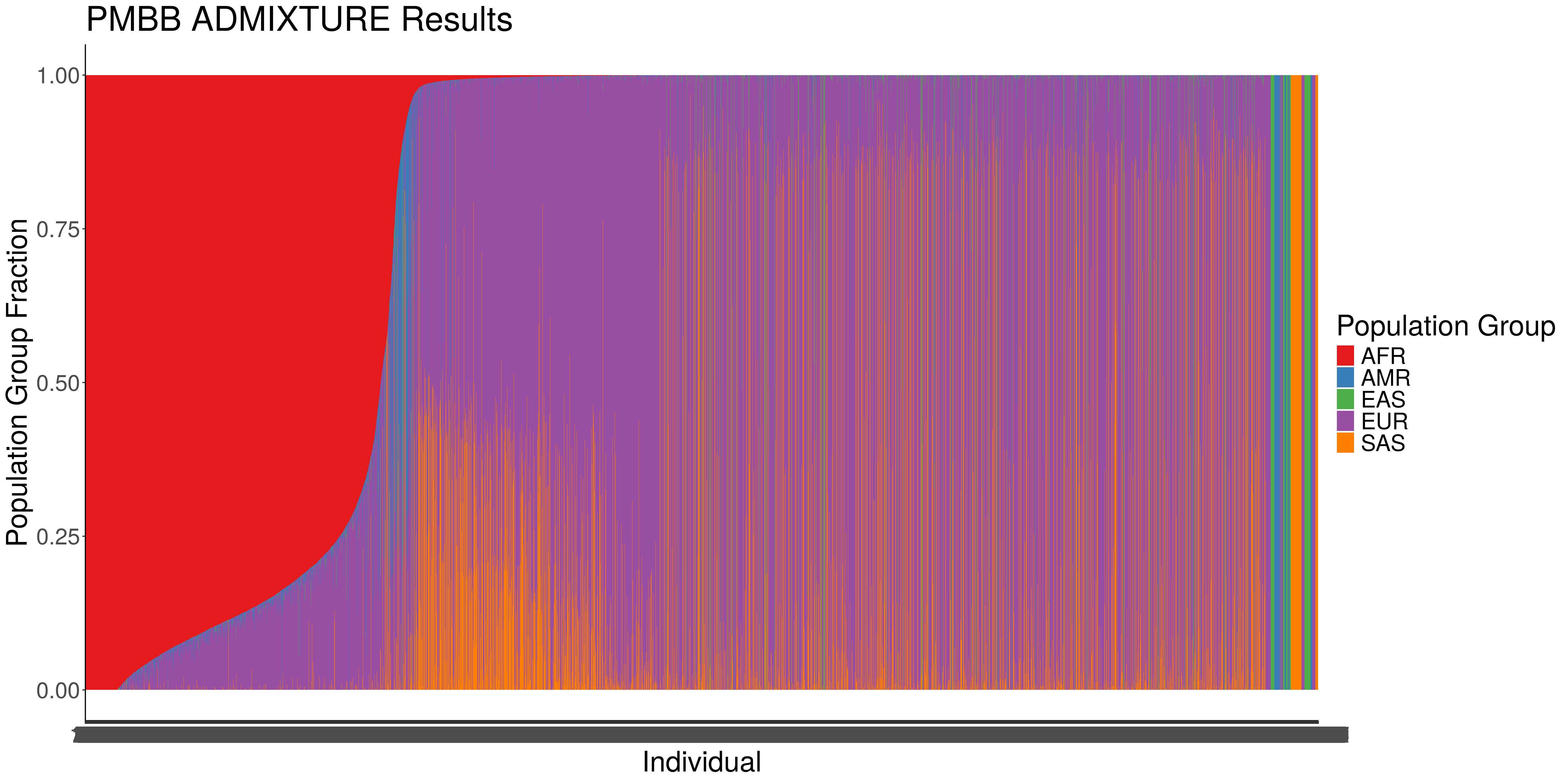

Supplement: S1 Fig — Vertical bars represent individual participants with stacked color bars representing a particular individual’s fractional composition of 1000 Genomes Project continental-level reference population groups (AFR: African reference population; AMR: Americas reference population; EAS: east Asian reference population; EUR: European reference population, SAS: south Asian reference population). (TIFF) [file pgen.1011727.s005.tiff]

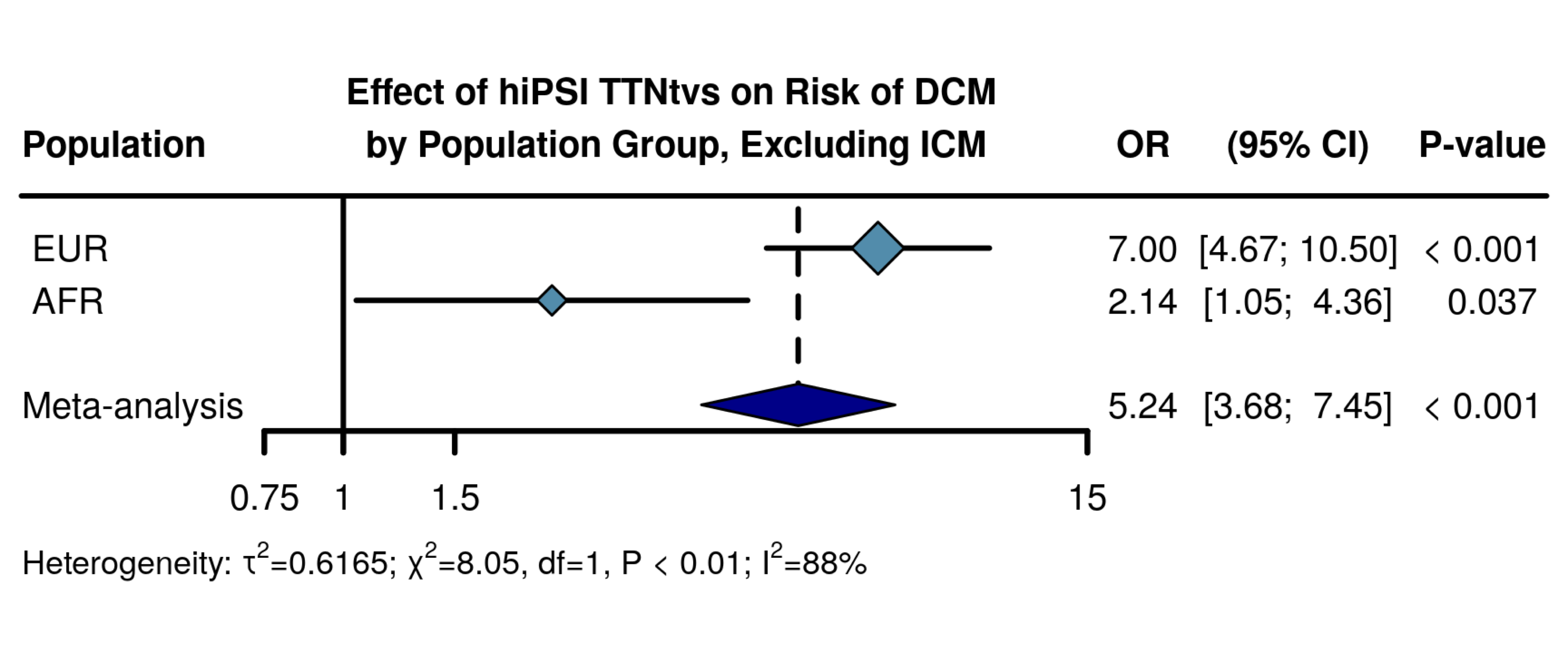

Supplement: S2 Fig — Logistic regression analysis of the association between hiPSI TTNtvs and DCM diagnosis among individuals genetically similar to the 1000 Genomes Project European and African reference population, and meta-analyzed. OR = odds ratio; CI = confidence interval; EUR = individuals genetically similar to the European reference population; AFR = individuals genetically similar to the African reference population; ICM = ischemic cardiomyopathy. (TIFF) [file pgen.1011727.s006.tiff]

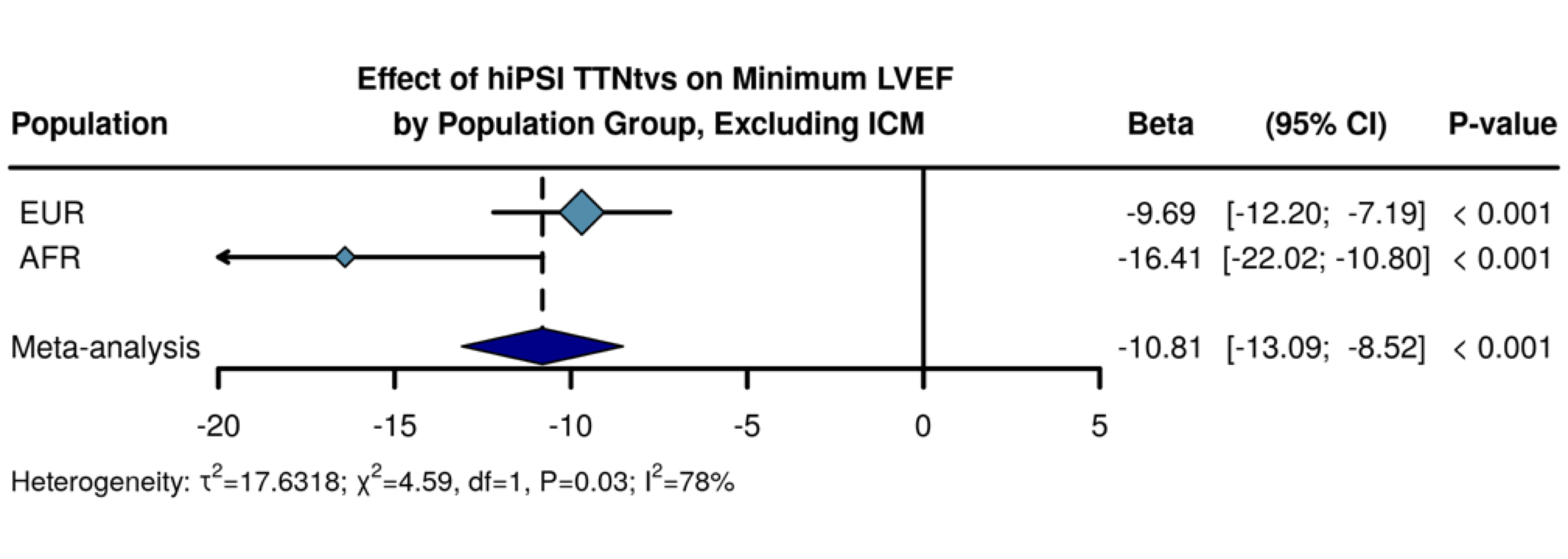

Supplement: S3 Fig — Linear regression analysis of the association between hiPSI TTNtvs on risk of decreased minimum LVEF stratified by genetic similarity to the 1000G EUR and AFR reference populations. CI = confidence interval; EUR = individuals genetically similar to the European reference population; AFR = individuals genetically similar to the African reference population; ICM = ischemic cardiomyopathy. (TIFF) [file pgen.1011727.s007.tiff]

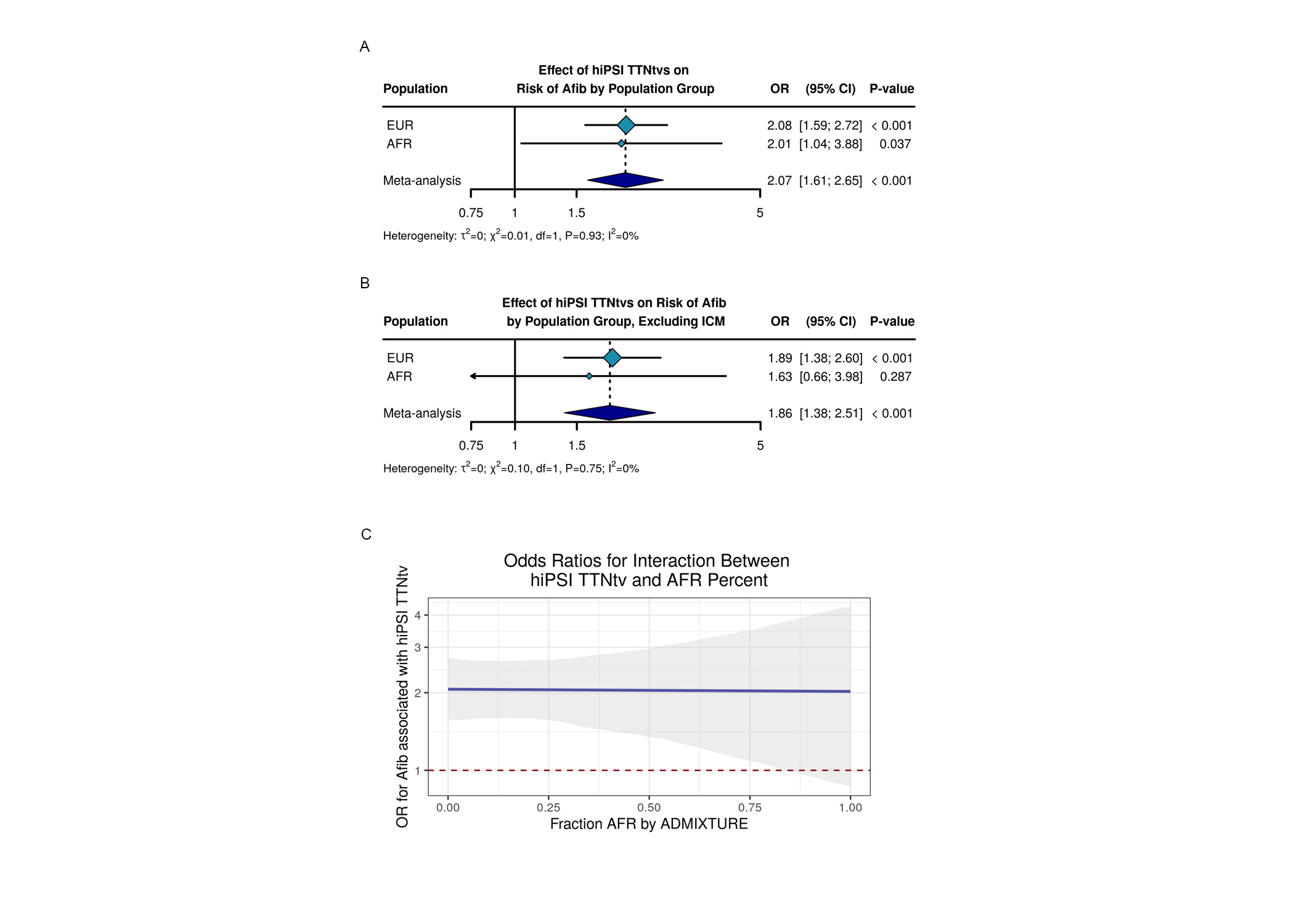

Supplement: S4 Fig — Logistic regression analysis of the association between hiPSI TTNtvs on risk of Afib stratified by genetic similarity to the 1000G EUR and AFR reference populations including (A) and excluding (B) individuals with ischemic cardiomyopathy with heterogeneity statistics included; (C) Logistic regression analysis of the interaction between hiPSI TTNtv and fraction AFR by ADMIXTURE where the dark blue line is the interaction effect estimate across the continuum of fraction of AFR, the grey shaded area are the 95% CIs, and the red dashed line is OR = 1. OR = odds ratio; CI = confidence interval; EUR = 1000G European reference population; AFR = 1000G African reference population; ICM = ischemic cardiomyopathy. (TIF) [file pgen.1011727.s008.tif]
