# Supplementary material for: Titin-Truncating variants predispose to dilated cardiomyopathy in populations genetically similar to african and european reference populations
Source: PLoS Genet. 2025 Jun 13;21(6):e1011727. doi: 10.1371/journal.pgen.1011727 (PMC12237270; doi:10.1371/journal.pgen.1011727)
Supplement: S1 Text — (DOCX) [file pgen.1011727.s009.docx]

Penn Medicine BioBank Banner Group Member List and Contribution Statements

PMBB Leadership Team

Daniel J. Rader, M.D., Marylyn D. Ritchie, Ph.D.

Contribution: All members contributed to securing funding, study design and oversight. All members reviewed the final version of the manuscript.

Patient Recruitment and Regulatory Oversight

JoEllen Weaver, Nawar Naseer, Ph.D., M.P.H., Afiya Poindexter, Khadijah Hu-Sain, Yi-An Ko, Ph.D.

Contributions: JW manages patient recruitment and regulatory oversight of study. NN manages participant engagement, assists with regulatory oversight, and researcher access. AP, KH, YK perform recruitment and enrollment of study participants.

Lab Operations

JoEllen Weaver, Meghan Livingstone, Fred Vadivieso, Stephanie DerOhannessian, Teo Tran, Julia Stephanowski, Monica Zielinski, Ned Haubein, Joseph Dunn

Contribution: JW, ML, FV, SD conduct oversight of lab operations. ML, FV, AK, SD, TT, JS, MZ perform sample processing. NH, JD are responsible for sample tracking and the laboratory information management system.

Clinical Informatics

Anurag Verma, Ph.D., Colleen Morse Kripke, M.S. DPT, MSA, Marjorie Risman, M.S..

Contribution: All members contributed to the development and validation of clinical phenotypes used to identify study subjects and (when applicable) controls.

Genome Informatics

Anurag Verma Ph.D., Shefali S. Verma, Ph.D., Yuki Bradford, M.S., Scott Dudek, M.S., Theodore Drivas, M.D., Ph.D.

Contribution: A.V., S.S.V. are responsible for the analysis, design, and infrastructure needed to quality control genotype and exome data. Y.B. performs the analysis. T.D. and A.V. provides variant and gene annotations and their functional interpretation of variants.
