# Supplementary material for: Titin-Truncating variants predispose to dilated cardiomyopathy in populations genetically similar to african and european reference populations
Source: PLoS Genet. 2025 Jun 13;21(6):e1011727. doi: 10.1371/journal.pgen.1011727 (PMC12237270; doi:10.1371/journal.pgen.1011727)
Supplement: Attachment — Submitted filename: Review.docx [file pgen.1011727.s010.docx]

The association of hiPSI TTNtvs on risk of DCM and other cardiac diseases has been found in individuals of European ancestry (EUR). So far, it has been a challenge to find the association in individuals of African ancestry (AFR), primarily due to the scarcity of datasets with a meaningful sample size. The authors investigated the effect of hiPSI TTNtvs on risk of DCM, Afib, and LVEF in diverse populations in the EHR-linked biobank, PMBB, to answer the question of whether individuals in other ancestries, in addition to EUR, reveals the expected effects. To assess risks of DCM, Afib, and LVEF by hipSI TTNtvs, a (large), diverse biobank, PMBB were used, alongside genetic distance (GD)-based grouping of individuals. GD is the Euclidian distance between a multi-dimensional vector for an individual, representing genotype of the person in the PCA space constructed with the 1000G dataset, and the centroid of the 1000G European individuals. Across diverse populations found in PMBB, their genotypes were projected in the PCA space, and using GD, 10 groups were identified as stratified groups, regardless of their ancestry background. Across the deciles, the association was examined with logistic regression for DCM and Afib, and with linear regression for LVEF with covariates such as age, sex, and/or PCs. The authors found no significant difference in effect of hiPSI TTNtvs on cardiac disease traits of interest across the decile groups.

The use of GD is the key idea for the author’s main conclusion on the association of the genetic predisposition with hiPSI TTNtvs for cardiac traits across diverse ancestries. The authors concluded with “(results), suggesting genetic background should not be considered when screening individuals for titin-related cardiovascular disease”.

Overall, the manuscript is well prepared and clear in the main points, along with the new results that are likely to be aligned with the recent work and that contrast with the results of prior work that were likely limited by the sample size. However, the rigor and the soundness of GD defined with the Euclidean distance in the PCA space as a metric for genetic similarity is unclear and subject to further investigation, which is the main concern, adding to the small sample sizes for other ancestries in particular including AFR. Without additional supporting materials and/or informative analyses to support the authors’ assertion, any conclusive assessment on findings of this study is difficult at this time. Some suggestions and comments are provided to address major concerns.

Here are my specific comments.

Major points

1. PCA has been widely used in population genetics, for example, for the adjustment of sub-population structure for GWAS, as a genetic distance in the study of polygenic score accuracy across the genetic ancestry continuum (Ding et al.), etc. However, PCA for population genetics is often used as black-box approach but complicated due to many complex factors influencing outcomes such as their dependency on the reference dataset for PCA space construction, genetic variants selected, the limitation of the linear transformation and the specific underlying algorithm potentially ignoring a certain aspect of patterns in a dataset, the difficulty to understand the consequence of relationship between the reference data and the target data, and so on (see for example, Elhaik, Eran. "Principal Component Analyses (PCA)-based findings in population genetic studies are highly biased and must be reevaluated." Scientific Reports 12.1 (2022): 14683). Additionally, in my opinion, the Euclidian distance estimated with PCA has not been thoroughly studied for its soundness as a metric for genetic similarity. Again, Ding et al. showed the use of GD for their main focus on the continuous variation of individual polygenic risk accuracy, but they did not address directly whether the metric is sufficiently good as the genetic similarity. Since the topic would be the beyond of the scope of this work, a better option would consider additional efforts to provide useful information that might indicate potential utilities, limitations, and challenges. For example, sharing all detailed information on the analyses for this study will allow replicated studies and will increase various efforts to find proper conditions empirically for successful applications. Providing more thoughts and additional analyses would be beneficial for understanding utility and potentials of the GD (with PCA) as genetic similarity measure. To address the concern, the suggested efforts I want to see include
   a. To refer prior studies, if exists, and provide more thoughts on the soundness and discussion for potential concerns on the use of the Euclidian distance for genetic similarity.
   b. To share the scripts and the analysis procedure in detail used for this manuscript as open access, allowing more information for a review as well as replicated studies expected to follow with different conditions using datasets from other biobanks or cohorts and using different PCA spaces constructed with different reference datasets.
   c. To provide additionally a kind of the sensitivity or secondary analysis. An example would be the use of the centroids of EAS or SAS, instead of that of EFR, but any sounding idea to address an issue with the subjective choice of the reference point in the PCA space or by showing the invariance of the metric measure in the PCA space in general would be helpful.
2. The authors need to provide thoughts on why the use of the PCA-based Euclidian distance might work for admixed populations for whom the underlying characteristics of admixed such as patterns in admixed haplotypes are likely to complicate the usefulness of the GD as a population descriptor across diverse populations comprising relatively homogeneous populations and admixed populations together.
3. The authors argued the deciles could represent diverse populations. However, it is not obvious whether the assumption is true or not. The authors need to elaborate more with additional information to support such an argument. Examples are
   a. Overall, the data is predominantly with individuals of EUR (about 70 %), alongside AFR (about 25 %). Previously, EUR was found with the expected association while AFR was not. One question is whether the results simply reflect individuals of EUR. If the comparison is made between the current results and new results obtained only with EUR, it would be more useful to understand the characteristics of the dataset with decile. The decile-wise comparison will be insightful.
   b. Another aspect critical for supporting the use of decile-based analysis is whether GD is invariant across two distinctive populations such as EUR and AFR. In other words, considering their distinctive locations in the space belonging to separated clusters (also considering their different genetic architecture), does the same value of GD represent the same extent of genetic similarity? One possible idea to examine for the issue is to compare their polygenic risk scores (or distributions) for DCM or other relevant cardiac diseases among individuals in the same decile. Any idea supporting the universal behavior of GD across different ancestries, e.g. AFR and EUR for representing their genetic predisposition against cardiac disease, can be considered for strengthening the main assumption for GD as genetic similarity.
   c. The authors need to describe details on how to generate deciles, to address a question for whether there exists heterogeneity or ancestry specific patterns within each decile with respect to EUR vs. others. Details might include the range of each decile, the largest GD value, the way to partition the data, etc, alongside a different kind of measures on decile dataset, in addition to the way the authors used, i.e. heterogeneity.
4. The authors discussed the possible reasons about the discrepancy on results between the prior work and the current one, in particular, regarding AFR. Considering the conflict conclusions from multiple studies in the literature, it is more informative if the authors present the comparison or a kind of summary (using the numbers for cases vs. controls, hiPSI TTNtvs carriers and/or characteristics of different datasets which likely affected outcomes) among studies, so the readers can have a better understanding on the circumstance and the potential significance of this work (or limitation).
5. The sample is unbalanced between cases and controls and their sizes are still not sufficiently large enough. The authors need to discuss why not to consider Firth logistic regression approach?

Minor points

1. For regression analysis, it is not clear why PCs are not used for covariates. See line 169, line 343. Please clarify this is correct and if so, any reason not to use PCs.
2. Provide, if possible, details on QC for genetic data such as sex errors, low sequence coverage with parameters used. (in lines 318-319)
3. Increase the font sizes for labels in Fig. 1 and Supplemental Fig. 1.
4. Add the label for gray (PMBB) in Fig. 1E, F, and G for the better readability.
5. At line 284, TTE appears without the definition.
