# Supplementary material for: Titin-Truncating variants predispose to dilated cardiomyopathy in populations genetically similar to african and european reference populations
Source: PLoS Genet. 2025 Jun 13;21(6):e1011727. doi: 10.1371/journal.pgen.1011727 (PMC12237270; doi:10.1371/journal.pgen.1011727)
Supplement: Attachment — Submitted filename: 11_02_24_PLOS_Gen_Detailed Response to Reviewers.docx [file pgen.1011727.s013.docx]

**Detailed Response to Reviewers**

Reviewer #1: Summary

DePaolo and colleagues used data from the population-based Penn Medicine Biobank (PMBB) to evaluate whether the effect of carrying a high percent spliced in (hiPSI) TTN truncating variant (TTNtv) on the risk of prevalent dilated cardiomyopathy (DCM) and related phenotypes varied across populations. They found that the effect of hiPSI TTNtvs on DCM risk, atrial fibrillation (Afib) risk, and left ventricular ejection fraction (LVEF) was similar across bins of genetic distance (GD), the metric that they chose to measure dissimilarity from European ancestry (EUR). Importantly, they also revised their conclusion in a prior study regarding the lack of effect of TTNtvs in African ancestry (AFR) populations, finding that hiPSI TTNtvs were associated with increased DCM and Afib risk and lower LVEF in AFR populations. While the results of this study are important, there are methodological issues that should be addressed, including undesirable properties of their GD metric, some technical statistical issues, and incomplete description of some methods. A revision addressing all issues below in which the GD metric was replaced or supplemented with continuous non-European ancestry proportions would certainly be worthy of further consideration.

**Reviewer 1 comment 1:** The authors should provide a reference detailing the design and comparative performance of the WeCall variant caller and explain why this was chosen over more standard pipelines based on the actively developed GATK software.

**Response:** The Github site for WeCall variant caller is now referenced in the manuscript. WeCall is fast, user friendly, has a high degree of fidelity, and has been implemented across the UKB for variant calling at scale (PMID 34662886 and 37308787). It has become a standard tool in variant calling.

Variant calling in the PMBB was performed in collaboration with Regeneron Genomics Center. The specific decision regarding the variant calling pipeline was determined internally by Regeneron for its computational efficiency and has been deployed across many datasets.

**Text change:** Reference added in line 312 on page 15.

**Reviewer 1 comment 2:** All statistical techniques described assume that individuals are unrelated, but there is no mention of whether this was verified. Please describe how cryptic relatedness between PMBB participants was assessed and addressed in analysis.

**Response:** Relatedness between the individuals was estimated using identify-by-descent. We removed the related individuals with pi-hat threshold of 0.25 which account for relatives up-to first cousins. This has now been added to the manuscript.

**Text change:** Lines 321-322 on page 15 now reads, “Relatedness between the individuals was estimated using identify-by-descent and related individuals with pi-hat threshold of 0.25 were excluded accounting for relatives up-to first cousins ([pmbb.med.upenn.edu](http://pmbb.med.upenn.edu)).”

**Reviewer 1 comment 3:** I would argue that the GD metric of Euclidean distance from the EUR centroid does not provide a relevant measure for this application. In particular, individuals in completely different regions of the ancestry PC space could have identical Euclidean distance from the EUR centroid, which means that individuals in genetically dissimilar populations (e.g., AFR and SAS) could be indistinguishable on this metric. Because individuals from dissimilar populations that may have dissimilar allele frequencies and disease risks can fall into the same grouping, this metric cannot provide an effective adjustment for population stratification without additional adjustment for other PCs. This lumping property also means that the metric does not allow estimation of interpretable ancestry-specific effects. Supplemental Table 1 and Supplemental Figure 1 show that this is more than a theoretical problem; ancestrally diverse groups were clearly lumped into the same GD category. Given these limitations, it is not entirely clear why the authors chose this metric instead of the more interpretable metric of non-European ancestry proportions (e.g., AFR, SAS, EAS, Native American) obtained from a model such as ADMIXTURE or directly from PCA. With these, the authors could determine whether and how an increasing proportion of each non-European ancestry modified the effect of carrying a hiPSI TTNtv, similar to the approach in reference 10. As this alternative approach would allow evaluation of the effect of ancestry on a continuum while minimizing residual confounding and enhancing interpretability, the authors should report results using it.

**Response:** We understand with the reviewer’s comment pertaining to the continuous measurement of genetic similarity to a population group. Our measure of genetic distance has some inherent flaws in its attempt to assess this. As the reviewer suggested, ADMIXTURE is a well-tested and accepted continuous metric of genetic similarity, and a variable we utilized in our group’s previous publication (PMID: 31216868). Therefore, we have removed all references to genetic distance in our manuscript and replaced it with analyses using ADMIXTURE as suggested above. This has altered the methods, results, and discussion substantially as discussed below.

Altogether, our findings produce a similar result to what we presented in the previous iteration of this manuscript, but with more clearly explainable findings. As you can see below, an increase in fraction of AFR population group as measured by ADMIXTURE is associated with a trend towards a smaller effect estimate, however the odds ratio remains significant and the widened error bars suggest a persistent limitation in power among non-EUR participants. This is not totally surprising as the PMBB remains overwhelming composed of participants genetically similar to the EUR reference population.

Thank you for suggesting this change.

**Text change:** The manuscript was changed throughout reflecting the altered analytic approach from genetic distance to integrating ADMIXTURE. Below are some examples.

**Lines 47-49 on page 2 (Abstract):** “Fraction of genetic similarity to the 1000 Genomes Project (1000G) African reference population was determined using ADMIXTURE analysis.”

**Lines 163-176 on pages 8-9**: “To further characterize the effect of TTNtvs across different populations groups, we modeled DCM as described by the interaction between hiPSI TTNtvs and proportion AFR by ADMIXTURE along with covariates including age, sex, and the first 5 genetic PCs (**Supplemental Table 1**). As shown in **Figure 2B**, though the hiPSI TTNtv effect estimate is smaller as an individual’s fraction of AFR population increases, the effect estimate remains robust. Notably, the 95% confidence intervals increase suggesting that this relationship remains limited by sample size among those with the highest proportion of AFR genetic similarity even with our increased cohort population. Taken together, these results suggest that TTNtvs are associated with DCM across diverse population groups, and specifically, there is no significant difference in hiPSI TTNtv effect on DCM risk related to genetic similarity to the 1000G AFR reference population either when considered as a dichotomized factor or continuous proportion.

**Lines 192-195 on page 10**: “These results were supported by our results using ADMIXTURE analysis to show robust effects of TTNtvs independent of fraction of AFR population group similarity.”

**Lines 335-341 on page 16:** “To quantify differences in genetic similarity to reference populations as a continuous variable among PMBB participants, proportional population group estimates were performed using ADMIXTURE (27). ADMIXTURE is a rapid and easily performed analysis that calculates the proportion of genetic similarity to reference populations using genotype data. These data are then algorithmically assessed based on the number of assumed reference populations provided to produce a proportion for each individual in the analytic cohort.

**Figure 2B on page 26** is now:

**Reviewer 1 comment 4:** The authors should include a description of how they defined “genetically similar” groups.

**Response:** Genetic similarity was based on genetic principal component analysis comparison to the 1000 Genomes Project reference populations. PMBB participants were projected into 1000G PCA space and then a Kernel Density Estimation for each continental-level reference population was employed to call genetic similarity to 1000G continental-level reference populations based on likelihoods. Participants were assigned to the population that they were most similar to. If no reference population had a high enough likelihood then the participant was labeled as “Unknown.” This has been added to the manuscript as described below.

**Text change:** Lines 326-333 on page 15 now reads, “To permit the comparison of the effect of TTNtvs across diverse populations, we used genetic principal components to group individuals into sub-populations based on genetic similarity to the 1000 Genomes Project (1000G) continental-level reference populations (26). Kernel Density Estimation was used to call participant population group based on likelihood similarity. While this approach is imperfect as it reinforces the artificial discretization of genetic diversity, it is necessary to create groups of individuals that share a component of common genetic variation in order to facilitate the evaluation of TTNtvs in these diverse populations.”

**Reviewer 1 comment 5:** It would be helpful to describe the logistic regression specifications used in detail. Based on the current descriptions, I have some concerns:

It appears that a model including age, sex, and TTNtv carrier status was fit separately in each GD bin and the results meta-analyzed. This approach is suboptimal for two reasons. First, the number of TTNtv carriers in each bin is likely small enough to warrant use of the Firth bias correction. Second, even if the Firth correction is unnecessary, the entire model could be fit with a single logistic regression in which intercept, age, sex, and TTNtv carrier status were interacted with GD bin, which would allow use of more reliable likelihood ratio tests and profile likelihood confidence intervals rather than the Wald-based procedures used for meta-analysis. Furthermore, using a single regression would allow model simplifications such as common sex and age effects that could potentially increase power.

**Response:** We agree with the reviewer’s concern, but also those concerns described **Reviewer 1 comment 3** where the shortcomings of using genetic distance was addressed. Please see the response to **Reviewer 1 comment 3** for further description as to how we have altered our analysis to include ADMIXTURE rather than GD from the European centroid. Because we utilized ADMIXTURE, we were able to use a continuous variable rather than bins of individuals of different genetic distances from the European centroid. We then modeled each of DCM, LVEF, and Afib as described by the interaction between hiPSI TTNtvs and fraction of genetic similarity to the 1000G AFR reference population to interrogate the effect of population group similarity as a continuous variable in addition to our analyses that were stratified by genetically similarity to a 1000 Genomes Project reference population.

**Text change:** Please see changes in response to **Reviewer 1 comment 3** above including **Figure 2B** that is shown in that response.

**Reviewer 1 comment 6:** It would be helpful to describe the logistic regression specifications used in detail. Based on the current descriptions, I have some concerns:

The functional form for age in the model should be described and justified.

**Response:** In this logistic regression, we used age-at-enrollment in the PMBB for age. We used this age in an effort to be consistent with previous analyses as it is the age we used in our first analysis of hiPSI TTNtvs on the risk of DCM (PMID: 31216868). We also used it because it is the most consistent way to discern an individual’s age in the PMBB as age at onset of disease is not consistently reported or easily derived for all individuals.

**Text change:** Lines 351-354 on page 17 now read: “Logistic regression was employed to evaluate the association of hiPSI TTNtvs with prevalent DCM adjusting for **age-at-enrollment** and sex. Linear regression was used to assess the effect of hiPSI TTNtvs on minimum left ventricular ejection fraction (LVEF) adjusting for **age-at-enrollment** and sex. Statistical analyses were performed using R (version 4.2.0).”

**Reviewer 1 comment 7:** It would be helpful to describe the logistic regression specifications used in detail. Based on the current descriptions, I have some concerns:

The estimated odds ratios presented in Supplemental Table 2 are not interpretable because they refer to a 1-unit change in GD, which is not possible as this variable has support on [0, 0.08] based on Figure 1. The odds ratios presented would be more informative if they corresponded to a 0.01-unit change.

**Response:** We agree with the reviewer’s comments here, though we have completely removed genetic distance from the analysis. We now use ADMIXTURE to determine the effect of TTNtvs on DCM risk across diverse populations. Please see our response to **Reviewer 1 Comment 3**.

**Text change:** Please see text change in **Reviewer 1 Comment 3**.

**Reviewer 1 comment 8:** For the linear regression with LVEF, it is unclear why meta-analysis was used instead of the more standard approach of fitting a single model in which intercept, age, sex, and TTNtv were interacted with GD bin. As the individual-level data are available, I would recommend the latter analysis approach to facilitate model simplification and more flexible inference.

**Response:** We have replaced GD analysis with ADMIXTURE and have included that as an interaction covariate in models to assess the effect of TTNtvs on LVEF. **Figure 3B** is shown below in our text change. We observed that there is no meaningful effect of proportion of AFR genetic similarity on TTNtv-derived risk of DCM, reduced LVEF, and Afib.

**Text change:** See response to **Reviewer 1 Comment 3** above where **Figure 2B** is shown; **Figure 3B on page 27** now appears as the following:

**Reviewer 1 comment 9:** The above comments regarding meta-analysis versus standard generalized linear model inference also apply to the genetically similar group analysis.

**Response:** To address the reviewer’s concerns, we have included the interaction term of fraction of genetic similarity to the AFR reference population and hiPSI TTNtv (AFR*hiPSI) as an analysis on each of DCM, LVEF, and Afib. We also included the analysis stratified by genetically similar subgroup to be consistent both with what our group has done previously to show that we were underpowered in that previous analysis, and also to remain consistent with what was most recently published by Shetty et al (PMID 39196186)

**Text change:** Please see text change to **Reviewer 1 Comments 3 and 8**, specifically **Figure 2B and 3B**.

Reviewer #2: Summary

The association of hiPSI TTNtvs on risk of DCM and other cardiac diseases has been found in individuals of European ancestry (EUR). So far, it has been a challenge to find the association in individuals of African ancestry (AFR), primarily due to the scarcity of datasets with a meaningful sample size. The authors investigated the effect of hiPSI TTNtvs on risk of DCM, Afib, and LVEF in diverse populations in the EHR-linked biobank, PMBB, to answer the question of whether individuals in other ancestries, in addition to EUR, reveals the expected effects. To assess risks of DCM, Afib, and LVEF by hiPSI TTNtvs, a (large), diverse biobank, PMBB were used, alongside genetic distance (GD)-based grouping of individuals. GD is the Euclidian distance between a multi-dimensional vector for an individual, representing genotype of the person in the PCA space constructed with the 1000G dataset, and the centroid of the 1000G European individuals. Across diverse populations found in PMBB, their genotypes were projected in the PCA space, and using GD, 10 groups were identified as stratified groups, regardless of their ancestry background. Across the deciles, the association was examined with logistic regression for DCM and Afib, and with linear regression for LVEF with covariates such as age, sex, and/or PCs. The authors found no significant difference in effect of hiPSI TTNtvs on cardiac disease traits of interest across the decile groups.

The use of GD is the key idea for the author’s main conclusion on the association of the genetic predisposition with hiPSI TTNtvs for cardiac traits across diverse ancestries. The authors concluded with “(results), suggesting genetic background should not be considered when screening individuals for titin-related cardiovascular disease”.

Overall, the manuscript is well prepared and clear in the main points, along with the new results that are likely to be aligned with the recent work and that contrast with the results of prior work that were likely limited by the sample size. However, the rigor and the soundness of GD defined with the Euclidean distance in the PCA space as a metric for genetic similarity is unclear and subject to further investigation, which is the main concern, adding to the small sample sizes for other ancestries in particular including AFR. Without additional supporting materials and/or informative analyses to support the authors’ assertion, any conclusive assessment on findings of this study is difficult at this time. Some suggestions and comments are provided to address major concerns.

**Reviewer 2 Comment 1:** PCA has been widely used in population genetics, for example, for the adjustment of sub-population structure for GWAS, as a genetic distance in the study of polygenic score accuracy across the genetic ancestry continuum (Ding et al.), etc. However, PCA for population genetics is often used as black-box approach but complicated due to many complex factors influencing outcomes such as their dependency on the reference dataset for PCA space construction, genetic variants selected, the limitation of the linear transformation and the specific underlying algorithm potentially ignoring a certain aspect of patterns in a dataset, the difficulty to understand the consequence of relationship between the reference data and the target data, and so on (see for example, Elhaik, Eran. "Principal Component Analyses (PCA)-based findings in population genetic studies are highly biased and must be reevaluated." Scientific Reports 12.1 (2022): 14683). Additionally, in my opinion, the Euclidian distance estimated with PCA has not been thoroughly studied for its soundness as a metric for genetic similarity. Again, Ding et al. showed the use of GD for their main focus on the continuous variation of individual polygenic risk accuracy, but they did not address directly whether the metric is sufficiently good as the genetic similarity. Since the topic would be the beyond of the scope of this work, a better option would consider additional efforts to provide useful information that might indicate potential utilities, limitations, and challenges. For example, sharing all detailed information on the analyses for this study will allow replicated studies and will increase various efforts to find proper conditions empirically for successful applications. Providing more thoughts and additional analyses would be beneficial for understanding utility and potentials of the GD (with PCA) as genetic similarity measure. To address the concern, the suggested efforts I want to see include

**Reviewer 2 Comment 1A:** To refer prior studies, if exists, and provide more thoughts on the soundness and discussion for potential concerns on the use of the Euclidian distance for genetic similarity.

**Response:** Genetic distance, as measured by the Euclidian distance from the European centroid, is not a broadly accepted continuous measurement of population difference. Therefore, and at the suggestion of Reviewer 1, we opted to use ADMIXTURE as a continuous measurement and have now included that in each of our analyses as referenced in response to **Reviewer 1 Comment 3**.

**Text change:** Extensive changes have been made throughout the manuscript and examples were included in the **Reviewer 1 Comment 3 Text Change.**

**Reviewer 2 Comment 1B:** To share the scripts and the analysis procedure in detail used for this manuscript as open access, allowing more information for a review as well as replicated studies expected to follow with different conditions using datasets from other biobanks or cohorts and using different PCA spaces constructed with different reference datasets.

**Response:** Please see response above in **Reviewer 2 comment 1A**. We will be happy to make our analysis code publicly available.

**Text change:** This statement has been added on Page 17 lines 359-360: “Code used in these analyses will be made publicly available at the time of publication.”

**Reviewer 2 Comment 1C:** To provide additionally a kind of the sensitivity or secondary analysis. An example would be the use of the centroids of EAS or SAS, instead of that of EFR, but any sounding idea to address an issue with the subjective choice of the reference point in the PCA space or by showing the invariance of the metric measure in the PCA space in general would be helpful.

**Response:** This is an interesting suggestion from the reviewer. Were we to continue to utilize genetic distance in our analysis, we would have pursued such a parallel study. However, because we altered our analysis to include ADMIXTURE as a continuous measurement of genetic similarity, and the use of population-stratified analyses as a sensitivity analysis, we did not include this change in our resubmission.

**Text change:** No specific changes were made.

**Reviewer 2 Comment 2:** The authors need to provide thoughts on why the use of the PCA-based Euclidian distance might work for admixed populations for whom the underlying characteristics of admixed such as patterns in admixed haplotypes are likely to complicate the usefulness of the GD as a population descriptor across diverse populations comprising relatively homogeneous populations and admixed populations together.

**Response:** We agree with the reviewer’s critique of genetic distance as a population descriptor. Therefore, we have utilized ADMIXTURE instead to perform our analysis. Please see the response to **Reviewer 1, Comment 3**.

**Text change:** Extensive changes have been made throughout the manuscript and examples were included in the **Reviewer 1 Comment 3 Text Change.**

**Reviewer 2 Comment 3A:** Overall, the data is predominantly with individuals of EUR (about 70%), alongside AFR (about 25%). Previously, EUR was found with the expected association while AFR was not. One question is whether the results simply reflect individuals of EUR. If the comparison is made between the current results and new results obtained only with EUR, it would be more useful to understand the characteristics of the dataset with decile. The decile-wise comparison will be insightful.

**Response:** This is an important point. First, for this reason, we have included a dichotomized analysis stratified by genetic similarity to the EUR and AFR reference population. Carrying a TTNtv is associated with an increased risk of a DCM diagnosis in both population groups (EUR: OR=6.16, 95% CI 4.34 to 8.74, *P*<0.001, AFR: OR=4.56, 95% CI 2.52 to 8.25, *P*<0.001). These results demonstrate overlapping 95% confidence intervals. Similar results were observed in the regression analysis of minimum LVEF and Afib.

Additionally, and separately from what we included in the previous iteration of this manuscript, we utilized ADMIXTURE to determine the effect of change in fraction of genetic similarity to the AFR reference population on the hiPSI TTNtv-associated risk of DCM. These results were demonstrated in **Figure 2B** that was included in **Reviewer 1 Comment 3 Text Change** which showed consistent effect across the range of AFR genetic similarity.

**Text change:** No specific change was made to the text.

**Reviewer 2 Comment 3B:** Another aspect critical for supporting the use of decile-based analysis is whether GD is invariant across two distinctive populations such as EUR and AFR. In other words, considering their distinctive locations in the space belonging to separated clusters (also considering their different genetic architecture), does the same value of GD represent the same extent of genetic similarity? One possible idea to examine for the issue is to compare their polygenic risk scores (or distributions) for DCM or other relevant cardiac diseases among individuals in the same decile. Any idea supporting the universal behavior of GD across different ancestries, e.g. AFR and EUR for representing their genetic predisposition against cardiac disease, can be considered for strengthening the main assumption for GD as genetic similarity.

**Response:** The reviewer makes important and helpful recommendations here, but the approach for this manuscript has changed substantially. Please see response to **Reviewer 1, Comment 3** regarding the change from using GD to ADMIXTURE.

**Text change:** No specific change was made.

**Reviewer 2 Comment 3C:** The authors need to describe details on how to generate deciles, to address a question for whether there exists heterogeneity or ancestry specific patterns within each decile with respect to EUR vs. others. Details might include the range of each decile, the largest GD value, the way to partition the data, etc, alongside a different kind of measures on decile dataset, in addition to the way the authors used, i.e. heterogeneity.

**Response:** We understand and agree with the reviewer’s recommendation here. Given our change in analysis approach, this has obviated the need to use decile-based analyses.

**Text change:** No specific change was made.

**Reviewer 2 Comment 4:** The authors discussed the possible reasons about the discrepancy on results between the prior work and the current one, in particular, regarding AFR. Considering the conflict conclusions from multiple studies in the literature, it is more informative if the authors present the comparison or a kind of summary (using the numbers for cases vs. controls, hiPSI TTNtvs carriers and/or characteristics of different datasets which likely affected outcomes) among studies, so the readers can have a better understanding on the circumstance and the potential significance of this work (or limitation).

**Response:** We appreciate the reviewer’s recommendation and have added the table below as a Supplemental Table.

**Text change: Supplemental Table 1** now appears as below:

| **Study Cohort** | **PMID** | **N cohort participants** | **N hiPSI TTNtv carriers (%)** | **N Patients with DCM (%)** | **AFR DCM OR (95% CI)** | **EUR DCM OR (95% CI)** |
| --- | --- | --- | --- | --- | --- | --- |
| PMBB | 31216868 | 10,289 | 142 (1.4%) | 570 (5.5%) | 1.8 (0.2–13.7) | 18.7 (9.1–39.4) |
| Geisinger | 31216868 | 61,040 | 359 (0.6%) | 595 (1.0%) | NA | 10.8 (7.0–16.0) |
| Jackson Heart Study | 31216868 | 2974 | 18 (0.6%) | 29 (1.0%) | 0 | NA |
| All of Us | 39196186 | 144,058 | 721 (0.5%) | 5,428 (3.8%) | 3.19 (1.97-5.15) | 3.85 (2.94–5.04) |

**Reviewer 2 comment 5:** The sample is unbalanced between cases and controls and their sizes are still not sufficiently large enough. The authors need to discuss why not to consider Firth logistic regression approach?

**Response:** Firth’s bias reduced logistic regression is often used for analyses with extremely rare event/case rates (ie <1%). In this present study, the rate of DCM was 2.4% and the rate of hiPSI TTNtvs was 1%. While these are relatively low, the overall sample size including controls is such that relatively low case rates should not invalidate our findings or bias our results. Furthermore, neither our previous work in this space (where 1.4% of individuals carried a hiPSI TTNtv), nor updated work by Shetty et al (PMID: 39196186) that we cite in our discussion and in which the case rate for DCM was 3.8% and hiPSI TTNtv was 0.5%, used Firth’s bias reduced logistic regression. For these reasons, we opted not to Firth’s bias reduced logistic regression.

**Text change:** No specific change was made to the text.

**Reviewer 2 comment 6:** For regression analysis, it is not clear why PCs are not used for covariates. See line 169, line 343. Please clarify this is correct and if so, any reason not to use PCs.

**Response:** PCs were used throughout all the regressions.

**Text change:** Line 351-352 on page 17 now read: “Logistic regression was employed to evaluate the association of hiPSI TTNtvs with prevalent DCM adjusting for age-at-enrollment, sex, and the first five genetic PCs.”

**Reviewer 2 comment 7:** Provide, if possible, details on QC for genetic data such as sex errors, low sequence coverage with parameters used. (in lines 318-319)

**Response:** We have added further details on the QC for genetic data as recommended.

**Text change:** Lines 313-316 on page 15 now reads: “Quality control exclusions included sex errors, high rates of heterozygosity (D-statistic > 0.4), low sequence coverage (<85% of targeted bases achieving 20X coverage), and genetically identified sample duplicates.”

**Reviewer 2 comment 8:** Increase the font sizes for labels in Fig. 1 and Supplemental Fig. 1.

**Response:** Font size for **Figure 1** has been increased. **Supplemental Figure 1** has been changed.

**Text change:** Figure 1 appears below:

**Reviewer 2 comment 9:** Add the label for gray (PMBB) in Fig. 1E, F, and G for the better readability.

**Response:** We appreciate the reviewer’s recommendation and have added a label for PMBB.

**Text change:** Please see response to **Reviewer 2 Comment 8**.

**Reviewer 2 comment 10:** At line 284, TTE appears without the definition

**Response:** We have updated the manuscript and the first time TTE is mentioned it is defined.

**Text change:** Line 180-186 on page 9 now read: “Using transthoracic echocardiography (TTE) data from all PMBB participants with LVEF values recorded, we found that carrying a hiPSI TTNtv was associated with a lower minimum LVEF among individuals genetically similar to the 1000G EUR and AFR reference populations (**Figure 3A,** EUR: Beta=-10.12%, 95% CI -12.76% to -7.48%, *P*<0.001, AFR: Beta=-12.59%, 95% CI -18.06% to -7.12%, *P*<0.001, Meta: Beta=-10.59%, 95% CI -12.96% to -8.21%, *P*<0.001; heterogeneity χ^2^=0.63, *P=*0.43, I^2^=0%).”
