# Supplementary material for: Titin-Truncating variants predispose to dilated cardiomyopathy in populations genetically similar to african and european reference populations
Source: PLoS Genet. 2025 Jun 13;21(6):e1011727. doi: 10.1371/journal.pgen.1011727 (PMC12237270; doi:10.1371/journal.pgen.1011727)
Supplement: Attachment — Submitted filename: PlosGen_review_Nov2024.docx [file pgen.1011727.s012.docx]

The authors revised the manuscript, primarily addressing the concern on the use of PCA-based genetic distance metric to rationalize the main conclusion for the implication of the multi-ancestry association analyses between hiPSI TTNtvs and DCM/Afib/LVEF for individuals of African ancestry. Defining ancestry with genetic population descriptors is a challenging but important problem in population genetics and other fields such as genetic medicine, and thus the authors’ effort to consider the problem carefully and to approach a solution sounding to the research community would be desirable for the current work. The research community will find more clues that will be available with bigger sample sizes and more diverse ancestry groups in future studies. Still, the main concerns such as the small sample sizes for non-European individuals, potential of selection bias intrinsically associated with the nature of datasets from EHR, and a possible overgeneralization of the outcome with the analysis using fraction of similarity to African ancestry by ADMIXUTURE remain. On the other hand, updated results based on an increased samples size from the prior work would be a good addition and, hopefully, a good resource for future following investigations.

There are a few minor comments related to mostly better readability and to be considered before the publication.

Minor comments

1. Line 127, 43,371 -> 43,731
2. Line 240, .(7) -> (7).
3. Line 279, the term of TTE appears here for the first time without explanation.
4. Provide more details on association analysis for Fig. 2B, Fig. 3B, and supplementary Fig. 4C. It sounds like *interplot* is the tool to be used. If so, state explicitly this with a brief description on the tool in Methods, so the reader can understand basic ideas, in addition to a current simple description that suggests simply the meaning of the approach.
5. Line 329-330, any parameters for defining boundaries?
6. The following sentence needs to be revised for better understanding.

Line 230-233: “The homogeneity of effect of when considering different proportions of AFR genetic similarity using ADMIXTURE analysis reduces the emphasis of result interpretation viewed through the lens of arbitrarily designated population groups.”
