# Supplementary material for: Titin-Truncating variants predispose to dilated cardiomyopathy in populations genetically similar to african and european reference populations
Source: PLoS Genet. 2025 Jun 13;21(6):e1011727. doi: 10.1371/journal.pgen.1011727 (PMC12237270; doi:10.1371/journal.pgen.1011727)
Supplement: Attachment — Submitted filename: 021825_PLOS_Gen_response.docx [file pgen.1011727.s014.docx]

**Detailed Response to Reviewers**

Reviewer #1: Summary

DePaolo and colleagues have submitted a responsive and thorough revision that completely addresses prior reviewer comments regarding their genetic distance metric. That the inferences remain unchanged is highly encouraging. While I applaud their efforts thus far, there are remaining issues that should be addressed to ensure that this potentially important work meets a high but attainable standard of rigor. I would find a revision in which these are addressed to be a compelling publication.

**Reviewer 1 comment 1:** Pi_hat is usually used to denote an estimate of the proportion of alleles shared IBD (pi), which is twice the kinship coefficient (phi). The kinship coefficient for first cousins is 0.0625, so pi for first cousins is 0.125, not 0.25. A pi_hat threshold of 0.25 would therefore not exclude first cousins or other third-degree relatives, which may not be stringent enough to eliminate problems due to cryptic relatedness. Moreover, pi_hat (= 2 * phi_hat) is an estimated IBD probability that will vary about its expectation of 0.125 for first cousins, potentially ranging as low as ~0.088 (see 10.1093/bioinformatics/btq559). The threshold for unrelated in this publication is phi_hat<0.044 (pi_hat<0.088); others (10.1002/gepi.21896) have used phi_hat<0.025 (pi_hat<0.05) for identifying unrelated individuals. Details on how this threshold was applied to select an unrelated subset should also be provided (e.g., PLINK algorithm). Finally, methods for obtaining an estimate of pi_hat and phi_hat should be described. Given the diverse nature of this sample, methods that account for distant relatedness due to ancestry (e.g., KING, PC-Relate) would be preferable, although methods assuming a sample from a single population would tend to overestimate relatedness (10.1016/j.ajhg.2015.11.022) and exclude unrelated individuals rather than underestimate it and include related ones.

**Response:** We appreciate the reviewer’s thorough review of the methodologies of kinship estimation. There are indeed a range of pi-hat values in the literature that are acceptable thresholds for cryptic relatedness above which individuals can be excluded. Several high-impact, contemporary genetic studies in large, diverse biobanks have identified pi-hat >0.25 as satisfactory threshold for exclusion of related individuals (PMIDs **39528825, 39528826, 38429522, 34140684, and 39363016). In addition to these external studies, we have utilized this threshold this pi-hat threshold across a range of studies undertaken in the PMBB, and recently described our methodology (PMID 36441943). Finally, in accordance with the reviewer’s recommendation, we have included a description in the manuscript and included it below.**

**Text change:** Lines 326-330 on page 15-16 now read: “Using PLINK (28), we identified and excluded cryptically related individuals (pi-hat >0.25) through identity by descent (IBD) analysis using the equation pi-hat  =  *P*(IBD = 2) + 0.5 × *P*(IBD = 1) where *P*(IBD = 2) is the probability of sharing two alleles identical by descent and *P*(IBD = 1) is the probability of sharing one allele identical by descent.”

**Reviewer 1 comment 2:** The description of the methods for population group descriptors is not sufficiently detailed. Kernel density estimation (KDE) is a very broad set of techniques in statistics involving many tuning parameters (e.g., bandwidth) that should be described. Presumably, KDE was used to estimate a multidimensional density for each 1000G reference population over the PC space, and the number of PCs considered (i.e., dimension of this density) should be clearly stated. How the resulting densities were used to assign a new individual to a population group should also be clearly stated. Was this simply the population for which that individual had highest density? Or was a Bayes classifier with non-uniform prior weights used? Without these details, it is difficult to evaluate the reasonableness of the overall approach for assigning individuals to ancestry groups, although the broad description seems to be on the right track and the assignments in Figure 1A-C look reasonable relative to the reference.

**Response:** We appreciate the reviewer’s recommendations for complete explanation of the use of KDE in the determination of population group. We have altered the text to meet the reviewer’s requests for a more detailed explanation as demonstrated below.

**Text change:** Lines 338-343 on page 16 now read: “Kernel Density Estimation (KDE) was then used to call participant population group based on likelihood similarity. Briefly, we used KDE to estimate multidimensional density for each 1000G reference population setting the number of dimensions to four (PC1 through PC4). PMBB participants were then assigned to population groups based on each participant’s highest likelihood relative to the 1000G reference population groups.”

**Reviewer 1 comment 3:** Several details regarding the ADMIXTURE analysis are missing. Were models with different numbers of latent populations (K) fit and compared (10.1007/978-1-0716-0199-0_4)? Were multiple runs from different starting points for each K performed and compared as recommended (10.1007/978-1-0716-0199-0_4 and 10.1093/bioinformatics/btw327)? How was the optimal K chosen?

**Response:** We once more appreciate the reviewer’s recommendation for a thorough explanation for the implementation of ADMIXTURE in the PMBB. We performed the analysis using the supervised ADMIXTURE procedure where the 1000G individuals, labeled using the five continental-level reference populations, were merged with the PMBB population and ADMIXTURE uses the labeled 1000G populations to learn from the reference populations. The number latent populations (k) was determined using the ADMIXTURE default settings of five-fold cross-validation. As the 1000G population contained only individuals labeled as one of five continental-level reference populations, the optimal k=5 was selected. No separate models were run for k or to select a k outside of these parameters. We have made the following subsequent changes to the description of the methods based on the reviewer’s recommendations above.

**Text change:** Lines 354-359 on page 17 now read: “In the present analysis, we performed a supervised ADMIXTURE analysis where 1000G individuals were labeled with their respective corresponding continental-level population group. Learning from those labeled 1000G individuals, fractions of continental-level population similarity were then assigned to PMBB participants using ADMIXTURE default setting and k (latent populations) = 5.”

**Reviewer 1 comment 4:** While use of age at enrollment is reasonable given the constraints of the data, it does require clarification of a few items in the outcome definitions. First, did diagnostic criteria for DCM and AFib consider only encounters prior to enrollment? This would be ideal as the logistic regression model then represents an implicit proportional odds survival model for DCM/Afib risk based on current status (at enrollment) data (see 10.1201/b16248, chapter 22). Second, over what period was the minimum LVEF determined, and how was this period related to age at enrollment?

**Response:** Thank you for pointing out this important issue and how it affects the analysis. In thinking about this more deeply, we determined that the most appropriate logistic regression analysis for the determination of risk associated with prevalent disease would be to use age-at-analysis as we considered cases of prevalent DCM or Afib irrespective of their timing relative to enrollment.

**Text change:** Lines 369-375 on page 17-18 now read: “Logistic regression was employed to evaluate the association of hiPSI TTNtvs with prevalent DCM adjusting for age-at-analysis, sex, and the first five genetic PCs. We utilized Firth’s bias-reduced logistic regression to model this effect due to the relative rarity of events using the logistf package (version 1.26.0) in R (31). Linear regression was used to assess the effect of hiPSI TTNtvs on minimum left ventricular ejection fraction (LVEF) determined prior or subsequent to enrollment, adjusting for age-at-analysis, sex, and the first five genetic PCs.”

**Reviewer 1 comment 5:** The authors should clarify in the manuscript that regressions used for the ancestry group analysis are completely stratified with the ancestry group-specific hiPSI TTNtv effects analyzed and combined using meta-analysis (although see my comments below about the issues with continued use of this approach). At the same time, the authors should clarify in the manuscript that the regressions including the interaction term with the global AFR proportion are not stratified. This entails additional assumptions regarding common effects of age at enrollment, sex, and ancestry PCs in AFR and non-AFR ancestries, so these are not different parameterizations of the ancestry-specific effect in the same model fit to the same sample. To obtain that, the authors would need to replace the AFR global ancestry proportion with a binary AFR = 1/EUR = 0 indicator and limit the analysis to individuals in just the AFR and EUR groups. All meta-analytic tests of heterogeneity and the combined TTNtv effect could then be obtained from nested models with and without the interaction.

**Response:** We appreciate the reviewer’s point and recognize that the way in which the analyses are currently described inadequately differentiates between the two logistic regression models. We have therefore clarified these analyses as described below.

**Text change:** Lines 375-381 on page 18 now read: “Logistic regression analyses of the effect of hiPSI TTNtvs were completely stratified by genetically similar population group, specifically EUR and AFR, and the population group hiPSI TTNtv effects were meta-analyzed. Logistic regression analyses of the effect of AFR proportion based on ADMIXTURE results, including the assessment of the interaction term between hiPSI TTNtv and AFR proportion, were not stratified by population group but only included EUR and AFR individuals included in the meta-analyzed analytic cohort.”

**Reviewer 1 comment 6:** If groups other than AFR and EUR are included in the model for the global ancestry proportion interaction, the TTNtv effect should also be allowed to vary by the proportions of other non-EUR global ancestries. Not doing so introduces a problem similar to the genetic distance metric in the last version of the manuscript, except now individuals who are nearly completely EUR, SAS, AMR, or EAS are all lumped into the reference group with the same assumed TTNtv effect. Figure 1A-C suggests a fair amount of admixture along other axes of ancestry, so there could be enough variation to include other non-EUR global ancestry proportions that vary in the sample (e.g., SAS, EAS, AMR) and their interactions with the TTNtv effect, which would solve the problem. Otherwise, limiting this analysis to the same combined sample as the meta-analysis of EUR and AFR groups would be a reasonable approach.

**Response:** The reviewer makes an important point here and we recognize the need to correct this analysis in our manuscript to properly describe the interaction of hiPSI TTNtv and AFR population. We have therefore altered our approach and included only EUR or AFR PMBB participants, or as the reviewer points out, only those that would be included in our meta-analyzed analysis. This has been stated in our **Methods** explicitly.

**Text change:** Please see the text change made in response to **Reviewer 1 comment 5**.

**Reviewer 1 comment 7:** The justification for not using Firth logistic regression in response to Reviewer 2’s comment is not entirely convincing. Prior work has shown that performance issues may arise with minor allele count <400 (10.1002/gepi.21742), a condition that applies here in the stratified meta-analysis. Using Firth correction also would not affect comparability with prior work if quasi-complete or complete separation were not an issue in this or any of these other studies. I agree with Reviewer 2 that using Firth logistic regression (and penalized likelihood inference on a unified model, as per my prior comments) would greatly increase confidence in the findings of this study with a relatively rare outcome and fairly low minor allele count.

**Response:** We appreciate the reviewer’s point with respect to using a Firth’s penalized logistic regression for the analyses and have implemented Firth’s in each of our logistic regression analyses. We also included this in the description of our methods as described below.

**Text change:** Lines 370-372 on page 17 now read: “We utilized Firth’s bias-reduced logistic regression to model this effect due to the relative rarity of events using the logistf package (version 1.26.0) in R (31).”

**Reviewer 1 comment 8:** The analysis in reference 10 relating to the effect of TTNtvs on DCM risk appears to have been case-control, not cross-sectional. This should be corrected.

**Response:** While the presentation of the data in reference 10 does appear to be case-control in nature, the actual study is cross-sectional in nature as it accounts for the genetic cause of DCM among all analyzed participants in the DCM Precision Medicine Study. The text of the language of the study, for example from the abstract, reads: “**Design:** Cross-sectional study enrolling patients with DCM who self-identified as non-Hispanic Black, Hispanic, or non-Hispanic White from June 7, 2016, to March 15, 2020, at 25 US advanced heart failure programs. Variants in 36 DCM genes were adjudicated as pathogenic, likely pathogenic, or of uncertain significance.” Additionally, the

**Text change:** No specific changes were made to the text.

**Reviewer 1 comment 9:** It would be helpful to also include a reference the cited UKB paper to show that WeCall has been widely employed.

**Response:** We appreciate the reviewer’s recommendation and have included that as a reference in our Methods section.

**Text change:** Lines 316-319 on page 15 now read “WeCall variant caller (v2.0.0, https://github.com/Genomicsplc/wecall) was employed for sequence alignment (GRCh38), variant identification, and genotype assignment as previously described (25).”

**Reviewer 1 comment 10:** It would be helpful to clearly describe in the manuscript whether the PCA and ADMIXTURE analyses were done on pooled 1000G and PMBB samples or whether PMBB samples were projected onto PC and ancestry spaces learned solely from 1000G samples. The response to reviewers seems to suggest that projection was used for PCA, in which case the method for centroid bias adjustment (10.1093/bioinformatics/btaa152), if any, should be described.

**Response:** For our PC analysis of PMBB participants, we used the smartpca program, part of the Eigensoft package. Using smartpca, we projected PMBB participants on to 1000G PC space to derive eigenvectors. Smartpca was run in fastmode and the number of output eigenvectors was set to 20. No centroid bias adjustment was employed.

Regarding ADMIXTURE, we performed a pooled analysis with 1000G participants labeled by continental-level population group. These labels were then used to learn and determine the different continental-level percentages for each PMBB participant. This was discussed further in response to **Reviewer 1, comment 3**.

**Text change:** Lines 333-338 on page 16 now read: “To permit the comparison of the effect of TTNtvs across AFR and EUR populations, we performed principal component (PC) analysis of PMBB participants. This was performed using the smartpca program, part of the Eigensoft package (https://github.com/DReichLab/EIG). Using smartpca, we projected PMBB participants on to 1000G PC space to derive eigenvectors. Smartpca was run in fastmode and the number of output eigenvectors was set to 20.”

**Reviewer 1 comment 11:** The ADMIXTURE plot in Supplemental Figure 1 raises some concerns. First, the global ancestry proportions do not appear to stack to 1 for a number of individuals. Second, it would be useful to see a plot for the 1000G reference individuals to inform the labeling used for latent subpopulations. This is especially relevant because the ACB, ASW, and AMR reference samples are representative of variable recent European-African or European-Native American(-African) admixture events in the Americas.

**Response:** The reviewer was correct – the global proportions did not stack to 1. One of the population groups in the original figure was not completely accounted for based on a coding error. That has been corrected, and the complete figure is now present as Supplemental Figure 1 and demonstrated below.

As to the question of running the same analysis on 1000G individuals to show the same plot, it would be impossible to replicate given the construction of our current ADMIXTURE analysis in the PMBB. Because we used a supervised analysis with labeled 1000G individuals to determine PMBB participant population proportions, we required having the 1000G labels known. We cannot run the same analysis using labeled 1000G individuals. We could consider running an analysis where PMBB participants were labeled and used as the reference individuals from which the 1000G population proportions could be derived, however this would obviously have weaknesses in terms of drawing conclusions for the 1000G individuals for whom population similarity has already been determined. The other consideration would be to run an unsupervised ADMIXTURE analysis on only the 1000G cohort, however that would be an entirely separate analysis and would not be translatable to what we show below. As these two options both lack translatability to the plot, and present analysis we show, neither is included in the revised manuscript.

**Text change: Supplemental Figure 1** now appears as demonstrated below:

**Reviewer 1 comment 12:** The conclusions in this manuscript do not apply to diverse populations such as AMR, SAS, or EAS, as appropriately noted by the authors in the limitations. However, the authors should limit the inferences to EUR and AFR (rather than “diverse populations”) more generally throughout the manuscript.

**Response:** We appreciate and agree with the reviewer’s concern with being overly broad in our conclusions, and we have made the corresponding changes throughout the text with examples shown below.

**Text change:** The manuscript title is now: “Titin-Truncating variants Predispose to Dilated Cardiomyopathy Populations Genetically Similar to African and European Reference Populations.” Lines 173-177 on page 9 now read: “Taken together, these results suggest that TTNtvs are associated with DCM across EUR and AFR population groups, and specifically, there is no significant difference in hiPSI TTNtv effect on DCM risk related to genetic similarity to the 1000G AFR reference population either when considered as a dichotomized factor or continuous proportion.” Similarly, lines 192-195 on page 10 now read: “Altogether, these results suggest that TTNtvs are associated with lower minimum LVEF across EUR and AFR population groups, and that this effect estimate is not impacted by a change in fraction of genetic similarity to the 1000G African reference population.”

Reviewer #2: Summary

The authors revised the manuscript, primarily addressing the concern on the use of PCA-based genetic distance metric to rationalize the main conclusion for the implication of the multi-ancestry association analyses between hiPSI TTNtvs and DCM/Afib/LVEF for individuals of African ancestry. Defining ancestry with genetic population descriptors is a challenging but important problem in population genetics and other fields such as genetic medicine, and thus the authors’ effort to consider the problem carefully and to approach a solution sounding to the research community would be desirable for the current work. The research community will find more clues that will be available with bigger sample sizes and more diverse ancestry groups in future studies. Still, the main concerns such as the small sample sizes for non-European individuals, potential of selection bias intrinsically associated with the nature of datasets from EHR, and a possible overgeneralization of the outcome with the analysis using fraction of similarity to African ancestry by ADMIXUTURE remain. On the other hand, updated results based on an increased samples size from the prior work would be a good addition and, hopefully, a good resource for future following investigations.

There are a few minor comments related to mostly better readability and to be considered before the publication.

**Reviewer 2 Comment 1:** Line 127, 43,371 -> 43,731

**Response:** This change was made in the updated manuscript.

**Text change:** Line 127 on page 6 now reads: “There were 43,731 participants in the analytic cohort (**Table 1**).”

**Reviewer 2 Comment 2:** Line 240, .(7) -> (7)

**Response:** This change has been made in the updated manuscript.

**Text change:** Lines 239-240 on page 12 now read: “Previously, we reported an OR of 18.7 (95% CI 9.1 to 39.4) for DCM among EUR individuals with hiPSI TTNtvs (7).”

**Reviewer 2 Comment 3:** Line 279, the term of TTE appears here for the first time without explanation

**Response:** TTE is first introduced on line 180 on page 9 which reads: “Using transthoracic echocardiography (TTE) data from all PMBB participants with LVEF values recorded…”

**Text change:** No specific changes were made to the text.

**Reviewer 2 Comment 4:** Provide more details on association analysis for Fig. 2B, Fig. 3B, and supplementary Fig. 4C. It sounds like *interplot* is the tool to be used. If so, state explicitly this with a brief description on the tool in Methods, so the reader can understand basic ideas, in addition to a current simple description that suggests simply the meaning of the approach.

**Response:** The reviewer is correct that this was written in an unclear manner in the previous version of the manuscript. In reality, *interplot* is not the tool to determine the effect of the interaction between hiPSI TTNtvs but rather the method to view it. This has been clarified in the latest revision and embellished based on the reviewer’s recommendation.

**Text change:** Lines 375-378 on page 18 now read: “Plots for the interaction of hiPSI TTNtv and proportion AFR were created using the *interplot* (version 0.2.3) package. *Interplot* is a user-friendly way to visualize changes in one variable in a two-way interaction term that is conditional on the value of another included variable a single regression statement.”

**Reviewer 2 Comment 5:** Line 329-330, any parameters for defining boundaries?

**Response:** We appreciate the reviewer’s question and have enhanced the description of our population group assignment based on KDE. Our primary parameter set for this analysis was the number of dimensions set to four (PC1 through PC4). We have changed the text accordingly as demonstrated below.

**Text change:** Lines 338-343 on page 16 now read: “Kernel Density Estimation (KDE) was then used to call participant population group based on likelihood similarity. Briefly, we used KDE to estimate multidimensional density for each 1000G reference population setting the number of dimensions to four (PC1 through PC4). PMBB participants were then assigned to population groups based on each participant’s highest likelihood relative to the 1000G reference population groups.”

**Reviewer 2 Comment 6:** The following sentence needs to be revised for better understanding. Line 230-233: “The homogeneity of effect of when considering different proportions of AFR genetic similarity using ADMIXTURE analysis reduces the emphasis of result interpretation viewed through the lens of arbitrarily designated population groups.”

**Response:** We appreciate and agree with the reviewer’s comment and have clarified the statement below.

**Text change:** Lines 230-234 on page 11 now read: “When proportion of AFR population similarity as determined by ADMIXTURE was utilized as a continuous variability in the present analysis, there was a consistent effect of hiPSI TTNtvs across the analytic cohort. This result reduces the emphasis placed on dichotomous genetic similarity especially in a substantially admixed population.”
