# Supplementary material for: Titin-Truncating variants predispose to dilated cardiomyopathy in populations genetically similar to african and european reference populations
Source: PLoS Genet. 2025 Jun 13;21(6):e1011727. doi: 10.1371/journal.pgen.1011727 (PMC12237270; doi:10.1371/journal.pgen.1011727)
Supplement: Attachment — Submitted filename: 042425_PLOS_Gen_response.docx [file pgen.1011727.s015.docx]

**Detailed Response to Reviewers**

Reviewer #1: Summary

DePaolo and colleagues have submitted a responsive and thorough revision that addresses nearly all of my comments on the prior revision. All issues itemized below should be addressable with minor changes to the manuscript.

**Reviewer 1 comment 1:** The authors should provide the PLINK version used (perhaps it was 1.07 based on the reference) and specify the method for selecting an unrelated subset (e.g., randomly select one relative from each pair).

**Response:** We appreciate the reviewer’s desire of completeness. We have therefore included the requested information as described below.

**Text change:** Lines 334-339 on page 16 now read: “Using PLINK (version 2.0) (29), we identified and excluded cryptically related individuals by randomly selecting one relative from each pair/group and excluding all others (pi-hat >0.25) through identity by descent (IBD) analysis using the equation pi-hat  =  *P*(IBD = 2) + 0.5 × *P*(IBD = 1) where *P*(IBD = 2) is the probability of sharing two alleles identical by descent and *P*(IBD = 1) is the probability of sharing one allele identical by descent.

**Reviewer 1 comment 2:** While the additional details are helpful, the authors should also provide information on the KDE software implementation, kernel type, and bandwidth selection technique.

**Response:** We appreciate the reviewer pushing us to describe the precise methodology we use for population group classification in the PMBB. It has provided opportunity for us to internally clarify exactly how this is performed in the PMBB pipeline. Population groups are assigned by a core service associated with the PMBB. We previously functioned under the assumption that this was performed by projecting PMBB participants into 1000 Genomes Project PC space. Instead, we have discovered that PMBB participants are projected into HapMap3 PC space and KDE is utilized to call population based on highest likelihood. HapMap3 populations are then mapped to 1000 Genomes Project continental-level reference populations.

In addition to this clarification, we have added the details requested by the reviewer as demonstrated below.

**Text change:** Lines 342-360 on pages 16-17 now read: “To compare the effect of TTNtvs across African (AFR) and European (EUR) populations, we conducted principal component analysis (PCA) on genetic data from PMBB participants using common variants. We employed the fastmode setting in smartpca (https://github.com/DReichLab/EIG) to generate 20 eigenvectors and projected PMBB participants onto HapMap3 (HM3) reference PC space. Population assignment utilized Kernel Density Estimation (KDE) with the KernSmooth package (version 2.23-26) as previously described (30). We constructed KDE models for each HM3 population using a 300×300 grid for both PC1/PC2 and PC3/PC4 dimensions. Bandwidth parameters were determined using the default "oversmoothed bandwidth selector" as described by Wand and Jones (31). For the PC1/PC2 KDE, the bandwidths by HM3 population included GIH (Gujarati Indians in Houston, Texas) = 0.007, CEU (Utah residents with Northern and Western European ancestry from the CEPH collection) = 0.008, ASW (African ancestry in Southwest USA) = 0.017, TSI (Toscans in Italy) = 0.001, MEX (Mexican ancestry in Los Angeles, California) = 0.006, and everything else = 0.008. For the PC3/4 KDE, the bandwidths by HM3 population included GIH = 0.0225, CEU = 0.004, ASW = 0.005, TSI = 0.004, MEX = 0.013, and everything else = 0.003. PMBB participants were assigned to population groups based on their highest likelihood scores relative to the HM3 reference populations which were subsequently mapped to 1000G continental-level reference populations.”

**Reviewer 1 comment 3:** The addition in lines 379-85 mostly addresses the comment, but I believe that this clarification should apply to both logistic *and linear* regressions. Please make that correction. Also, please specify the meta-analytic method used (e.g., inverse variance weighted fixed effects).

**Response:** We have added the relevant details below.

**Text change:** Lines 395-399 on page 19 now read: “**Logistic and linear regression** analyses of the effect of hiPSI TTNtvs were completely stratified by genetically similar population group, specifically EUR and AFR, and the population group hiPSI TTNtv effects were meta-analyzed **using the inverse variance weighted method with fixed effects**. **Logistic and linear regression analyses** of the effect of AFR proportion based on ADMIXTURE results, including the assessment of the interaction term between hiPSI TTNtv and AFR proportion, were not stratified by population group but only included EUR and AFR individuals included in the meta-analyzed analytic cohort.”

**Reviewer 1 comment 4:** This still needs to be corrected. While the study was cross-sectional with respect to the probands, all of whom had DCM, the supplemental results on the association of TTNtvs with DCM risk in eAppendix 2 were based on a comparison of probands (cases) to *external population controls* from 1000 Genomes. As all probands in the cross-sectional sample analyzed in the main text had DCM, there would be no way to evaluate the association of TTNtvs with DCM risk in just probands; doing so would require pairing external controls with these cases, as in eAppendix 2. The authors of reference 11 also make explicit references to the case-control nature of the TTNtv analysis and its reliance on external controls in eAppendix 2 with statements such as:
-- “The odds of harboring a TTN pLOF variant were modeled as a function of case status (DCM proband or 1000 Genomes control)…”
-- “This retrospective formulation of the logistic regression was chosen because it more closely reflects the sampling design, which was stratified on both case status and ancestry so that only whether an individual was found to harbor a TTN pLOF variant was an unrestricted random quantity.”

**Response:** We appreciate the reviewers identifying this difference and have changed it below.

**Text change:** Lines 101-105 on page 5 now read: “Recently, a **case-control** analysis of individuals with DCM demonstrated a statistically significant but attenuated effect of predicted loss-of-function (pLOF) variants in *TTN* among AFR individuals compared to EUR individuals, potentially underscoring the limitations of applying genetic understanding of disease derived from a single ancestry group to diverse populations”

**Reviewer 1 comment 5:** The lack of centroid bias adjustment in PC projection may result in under-adjustment for ancestry differences (10.1093/bioinformatics/btaa152, 10.1093/bioinformatics/btaa520), although shrinkage may be relatively small for the top 4-5 PCs when using the entire 1000 Genomes reference (10.1093/bioinformatics/btaa152, 10.1093/bioinformatics/btaa520). I would suggest that this be included as a potential limitation mitigated by the likelihood that shrinkage was small based on data in the references above.

**Response:** We have now included this as a limitation in our manuscript.

**Text change:** Lines 288-290 on page 14 now reads: “Finally, we did not perform a centroid bias adjustment in the PMBB PC projection that may have resulted in under-adjustments for differences in population.”

**Reviewer 1 comment 6:** The corrected ADMIXTURE plot seems to indicate a degree of genetic similarity with SAS reference populations much higher than that reported by the authors in Table 1 and elsewhere for the entire cohort (10.3390/jpm12121974), all concentrated within individuals with low genetic similarity to AFR. The source of this phenomenon should be investigated and explained to the reader. One possible explanation is that ADMIXTURE's optimization algorithm terminated at a local maximum of the likelihood; the authors should try rerunning ADMIXTURE at least 10 times using different seeds and comparing the results to confirm that this is indeed a global maximum, as recommended in my prior comments and by the ADMIXTURE authors themselves (10.1007/978-1-0716-0199-0_4).

**Response:** Per the reviewer’s recommendation, we reran ADMIXTURE 10 times with different seeds and reproduced the same ADMIXTURE plot shown below: We have also commented on it in the main text as demonstrated below.

Here is the original plot of Admixture results in the PMBB:

Here are the 10 reruns of Admixture:

As you can observe, the general %SAS remains stable, though there is some fluctuation, and is largely grouped within the non-AFR population, as the reviewer points out.

To demonstrate the variance by PMBB participant, we plotted the variance and mean with each dot representing a unique individual below:

As observed above, the variance is highest at lower proportions of %SAS with these values corresponding to the fluctuations observed in the plots but ultimately not having any impact on the conclusions observed in the manuscript.

**Text change:** Lines 146-149 on page 8 now read: “Notably, variances in percentage similarity to specific populations may overestimate overall fractions of similarity in this plot due to ADMIXTURE's optimization algorithm terminating at a local maximum of the likelihood as described previously (20).”

**Reviewer 1 comment 7:** The AFR variable is the fraction of genetic similarity to the AFR reference population, which has support on [0, 1]. Because the coefficient on AFR and the interaction with hiPSI are effects for a 1-unit change in AFR, they are therefore a bit hard to interpret. The authors could rescale these coefficients to be for a change of 0.1 in the fraction of genetic similarity, which would be more interpretable without changing any inferences or plots.

**Response:** We have made the relevant changes to **Supplemental Table 2-4** such that the change in percent AFR is representative of a 0.1 change in percent AFR. The tables are included below. We have also made the relevant changes in the description of the tables.

**Text change: Supplemental Tables 2-4** are shown below:

**Supplemental Table 2: Effect of hiPSI *TTN* truncating variants, a 0.1 change in fraction of genetic similarity to the AFR reference population, and the interaction of the two covariates in logistic regression analysis of DCM risk accounting for age, sex, and the first five genetic PCs.**

| **Regression Term** | **DCM Odds Ratio** | **LCB** | **UCB** | **P** |
| --- | --- | --- | --- | --- |
| hiPSI | 6.20 | 4.38 | 8.79 | 9.13E-25 |
| AFR (0.1) | 1.26 | 0.79 | 1.99 | 0.33 |
| hiPSI:AFR (0.1) | 0.96 | 0.90 | 1.05 | 0.43 |

**Supplemental Table 3: Supplemental Table 3: Effect of hiPSI *TTN* truncating variants, a 0.1 change in fraction of genetic similarity to the AFR reference population, and the interaction of the two covariates in linear regression analysis of change in LVEF accounting for age, sex, and the first five genetic PCs.**

| **Regression Term** | **Beta LVEF** | **LCB** | **UCB** | **P** |
| --- | --- | --- | --- | --- |
| hiPSI | -10.10 | -12.83 | -7.37 | 4.53E-13 |
| AFR (0.1) | -0.87 | -3.33 | 1.58 | 0.49 |
| hiPSI: AFR (0.1) | -0.33 | -0.99 | 0.32 | 0.32 |

**Supplemental Table 4: Effect of hiPSI *TTN* truncating variants, a 0.1 change in fraction of genetic similarity to the AFR reference population, and the interaction of the two covariates in logistic regression analysis of Afib risk accounting for age, sex, and the first five genetic PCs.**

| **Regression Term** | **Afib OR** | **LCB** | **UCB** | **P** |
| --- | --- | --- | --- | --- |
| hiPSI | 1.86 | 1.44 | 2.42 | 4.68E-05 |
| AFR (0.1) | 1.16 | 0.89 | 1.51 | 1.00 |
| hiPSI: AFR (0.1) | 0.99 | 0.92 | 1.08 | 0.21 |

**Reviewer 1 comment 8:** The phrase “…the effect estimate is not significantly associated with an interaction between hiPSI and AFR…” is a bit confusing. The authors should say that either (1) there was no evidence of interaction between hiPSI and AFR or (2) the hiPSI effect estimate was not significantly associated with the fraction of genetic similarity to AFR.

**Response:** We have altered the verbiage as suggested.

**Text change:** As an example, lines 169-172 on page 9 now reads: “As shown in **Figure 2B**, the effect of hiPSI TTNtv on DCM risk is consistent across the spectrum of AFR fraction, and **the hiPSI effect estimate was not significantly associated with the fraction of genetic similarity to AFR** (*P*=0.43)”

Reviewer #2: Summary

Based on this revision, I do not have major issues and would like to suggest the manuscript is publishable. Minor comments are provided as follows for improving readability.

**Reviewer 2 comment 1:** Line 159, “P=008” seems to be typo.

**Response:** The reviewer is correct. The *P*-value for heterogeneity has been corrects and now reads as shown below.

**Text change:** Line 161 on page 8 now reads: “heterogeneity χ^2^=2.97, *P=*0.08, I^2^=66%.

**Reviewer 2 comment 2:** Line 340-341, “Using smartpca, we projected PMBB participants on to 1000G PC space to derive eigenvectors” sounds inaccurate. Eigenvectors in PCA are PC vectors and, as the author indicated, it is the projection of a sample data on those eigenvectors obtained with 1000G data, instead of the derivation of eigenvectors.

**Response:** We appreciate the reviewer’s critique and have made the suggested change.

**Text change:** Lines 344-346 on page 16 now read: “We employed the fastmode setting in smartpca (https://github.com/DReichLab/EIG) to generate 20 eigenvectors and projected PMBB participants onto HapMap3 (HM3) reference PC space.”
